# A Convolutional Mixer-Based Deep Learning Network for Alzheimer’s Disease Classification from Structural Magnetic Resonance Imaging

**DOI:** 10.3390/diagnostics15111318

**Published:** 2025-05-23

**Authors:** M. Krithika Alias Anbu Devi, K. Suganthi

**Affiliations:** School of Electronics Engineering, Vellore Institute of Technology, Chennai 600127, India; krithika.anbudevi@vit.ac.in

**Keywords:** Alzheimer’s disease, convolutional neural network, deep learning, MRI, SMOTEENN

## Abstract

**Objective:** Alzheimer’s disease (AD) is a neurodegenerative disorder that severely impairs cognitive function across various age groups, ranging from early to late sixties. It progresses from mild to severe stages, so an accurate diagnostic tool is necessary for effective intervention and treatment planning. **Methods:** This work proposes a novel AD classification architecture that integrates depthwise separable convolutional layers with traditional convolutional layers to efficiently extract features from structural magnetic resonance imaging (sMRI) scans. This model benefits from excellent feature extraction and lightweight operation, which reduces the number of parameters without compromising accuracy. The model learns from scratch with optimized weight initialization, resulting in faster convergence and improved generalization. However, medical imaging datasets contain class imbalance as a major challenge, which often results in biased models with poor generalization to the underrepresented disease stages. A hybrid sampling approach combining SMOTE (synthetic minority oversampling technique) with the ENN (edited nearest neighbors) effectively handles the complications of class imbalance issue inherent in the datasets. An explainable activation space occlusion sensitivity map (ASOP) pixel attribution method is employed to highlight the critical regions of input images that influence the classification decisions across different stages of AD. **Results and Conclusions:** The proposed model outperformed several state-of-the-art transfer learning architectures, including VGG19, DenseNet201, EfficientNetV2S, MobileNet, ResNet152, InceptionV3, and Xception. It achieves noteworthy results in disease stage classification, with an accuracy of 98.87%, an F1 score of 98.86%, a precision of 98.80%, and recall of 98.69%. These results demonstrate the effectiveness of the proposed model for classifying stages of AD progression.

## 1. Introduction

AD is the prevailing cause of dementia, involving the degeneration of brain tissues and nerve cells, disrupting normal brain function. It is a complex neurodegenerative disorder associated with a decline in cognitive functions. This public health concern affects 55 million individuals in low- and middle-income groups worldwide. It is the seventh leading cause of increased mortality rates, and the World Health Organization (WHO) reports an annual increment of about 10 million cases [1]. Families spend over USD 1.3 trillion in a year on medical care and treatment, which has a significant financial impact and elevates stress and discomfort for caregivers.

AD is not a natural part of aging. About 6 million Americans [2,3] under the age of 65 are afflicted by this disease. This early-onset type is called younger-onset Alzheimer’s disease. At the early stage, neurofibrillary tangles and amyloid plaques are formed in the brain. Later, brain atrophy reduces the volume of the hippocampus and entorhinal cortex. Developing effective and clinically relevant early detection methods requires distinguishing prodromal AD, also referred to as mild cognitive impairment (MCI).

The earliest stage of cognitive decline is represented by MCI, which is divided into late mild cognitive impairment (LMCI) and early mild cognitive impairment (EMCI). EMCI individuals might experience cognitive difficulties without a significant impact on daily activities, while LMCI is characterized by a more noticeable memory decline compared to EMCI. Distinguishing between EMCI and LMCI sub-types for diagnosis often involves using memory scores, although this approach may lead to lower specificity and potential misclassifications. Those with EMCI symptoms have a lower likelihood of progressing to the AD stage. There is a growing emphasis on identifying subtle changes among MCI patients, particularly in the LMCI stage, as this phase may present an opportune window for therapeutic interventions aimed at altering disease progression [4]. Clinicians increasingly rely on computer-aided diagnostic tools to monitor disease progression and inform treatment strategies effectively. Different imaging modalities are available for diagnosing AD.

In computed tomography–positron emission tomography (CT-PET) neuroimaging, a radioisotope is injected into the human body to track regional brain metabolism. However, patients with hyperglycemia may experience compromised accuracy due to potential chemical imbalances. Magnetic resonance imaging (MRI) generally offers superior contrast compared to computed tomography (CT) imaging modalities [5,6]. The multimodality approach poses challenges in obtaining data from multiple imaging modalities for a single patient. In this work, non-invasive T2-weighted high-tissue-contrast MRI images are utilized to classify the different stages of AD. Machine learning and deep learning are two artificial intelligence approaches for diagnosing the progression of AD. Neuroimaging techniques [7] provide enhanced accuracy in classifying the different stages of AD when combined with machine learning techniques. Implementing a machine learning [8] approach needs critical preprocessing steps including feature selection, dimensionality reduction, and classifier design. In contrast, deep learning models [9,10] can automatically extract features from MRI scans. Deep learning (DL) has a hard goal of achieving high accuracy and reasonable training time. These are particularly responsive to the initial values of weights, as highlighted in [8]. Inadequate weight initialization can significantly slow down convergence. Proper weight initialization mitigates issues like exploding or vanishing activation outputs during training. Our research adds to a noteworthy trend line that was initiated by MobileNet [11] and Xception [12], indicating that separable convolutions may be used to replace convolutions in any convolutional model, whether for 1D or 2D data, to provide a model that is simultaneously smaller, more affordable to operate, and slightly better in terms of performance. Both strong theoretical underpinnings and encouraging experimental data support this tendency. Our proposed model is different from vision mixer [13] and covmixer [14]. Vision mixer architecture uses an MLP mixer with a spatial and channel mixing concept and tokenizes the input into a fixed size. A convolutional mixer uses multiple depthwise and pointwise convolution with residual connections. Our model simplifies this by using depthwise separable convolutions only in deeper layers, reducing trainable parameters and FLOPs, as reflected in our efficiency metrics. It operates on the complete resolution of feature maps and preserves spatial information throughout the layer.

This research focuses on the following key aspects:

- Optimizing neural network parameters: This includes selecting appropriate weight initialization methods. It reduces convergence and establishes stable learning biases within the network.
- Reducing trainable parameters: This approach aims to minimize the number of trainable parameters and computational complexity (reduces the number of floating-point operations).
- Anatomical feature detection: Potential changes in anatomical features across different classes are detected using ASOP pixel attribution method This method enhances understanding of how neural networks interpret and distinguish features in an input image.
- Handling class imbalance: The class imbalance within the dataset can lead to biased learning outcomes if not balanced through a balancing technique. The synthetic minority oversampling technique (SMOTE) combines with edited nearest neighbors (ENN) to balance the distribution of classes in datasets, which overcomes the under-fitting predicament.

Section 2 describes the contribution of related works. The proposed model are described in Section 3. An ablation study is presented in Section 4 to select the hyper-parameter for the proposed model. The formulation of performance metrics is in Section 5. Results are highlighted in Section 6. Section 7, Section 8 and Section 9 summarized the proposed research and discuss future work.

## 2. Related Works

Automatic disease detection represents a formidable challenge within the medical domain. Deep learning methodologies are pivotal in disease diagnosis, serving as invaluable tools to aid healthcare professionals in predicting disease presence and progression from critical anatomical regions within the human body. Several pertinent studies focusing on Alzheimer’s disease classification illustrate advancements in this field. This section emphasizes recent advancements in AD classification, focusing on convolutional neural network (CNN) architecture, MRI modality integration, and addressing class imbalance challenges. In [15], Mcnemars test distinguished the nominal dependent variables between two related groups. It assessed the statistical significance and performance of EfficientNetB0 architecture. In [16], a CNN end-to-end-framework model achieved accuracy of 97.5% in a multiclass classification task. In [17], a CNN model was optimized with the spider monkey optimization algorithm, and the SMOTE oversampling technique was employed to balance the data distribution in all stages.

In [18], the inception V3 model was used as the base model. The green fire blue filter was added in the preprocessing layer to enhance image quality, resulting in a testing accuracy of 98.68% and a validation accuracy of 97.81%. The triplet-loss function is implemented in a Siamese convolutional neural network [19], utilizing the VGG-16 architecture to extract features for performing four-way classification of AD. In [20], a saliency activation map is used for predicting specific classes. The Keras pretrained application model ResNet50v2 was implemented along with the SMOTE oversampling technique for multiclass classification, achieving an accuracy of 91.84%. In MobileNet [21], the notable feature is the use of depthwise separable convolutions, and it provides the multiclass classification accuracy of 96.6%. A curvelet transform-based deep convolutional [22] detects the MRI features in terms of a low level, which reduces the noise amplification. The unequal space FFT and wrapping-based method generate coefficients. The wrap-based method also requires less computational time. Kurtosis thresholding removes all types of noise in the MRI and improves the quality of images.

DEMNET [23] uses a residual and inception network as the base classifier model alongside three convolutional layers with ReLU and batch normalization. This classifies four stages of AD from the Kaggle open access series of imaging studies (OASIS) dataset, with SMOTE used to balance the dataset. In ADDNET [24], the synthetic minority oversampling tomek link (SMOTE-TOMEK) oversampling algorithm addresses the class imbalance problem in the Kaggle dataset, and the CNN model is fine-tuned to reduce the computational cost. In [25], 2D and 3D brain structural MRI features are extracted using 2D and 3D convolutional operation, and a transfer learning VGG 19 model is fine-tuned to classify the different stages of disease. In LeNET [26], a leaky rectified linear activation function, sigmoid, and batch normalization are implemented, achieving an accuracy of 83.7%.

In [27], a dense block is added to the deep-layer VGG 19 model to overcome the vanishing gradient problem. The complexity introduced by bridge connections is minimized by implementing depthwise convolution instead of normal convolutional operations. The multivariate T2 hoteling statistical test [28] distinguishes features variation in the AD and CN stage. Each patch is fed into the single CNN, and its size is decreased to enhance the network’s performance. Initial layers of Alexnet [29] are altered according to the dataset and extract the crucial features. SVM, K-nearest neighbor, and the random forest classifier are used for classification. In [30], a CNN model with an inception block extracted the gray matter volume to identify the AD. This research primarily focuses on improving existing convolutional neural network (CNN) architectures. The dataset utilized in this study is relatively small, and the large-scale transformer models are not discussed. Transformer-based architectures generally require vast amounts of data to outperform CNNs, especially when trained from scratch [31]. Moreover, the computational complexity of transformer models is considerably higher than that of CNNs [32]. Transformer models cannot outperform CNNs in all image analysis, particularly with low-resolution medical images [33]. Therefore, the present work prioritizes CNN-based approaches, which remain highly effective for the given dataset size. The proposed work is unique from existing models, as the following details:

- The existing CNN models are often trained with a large number of trainable parameters. In this research, the application of convolutional mixer architecture reduces the number of trainable parameters, thereby reducing computational demands without compromising performance.
- Existing convolutional mixers use multiple depthwise and pointwise convolution with residual connections. Our model simplifies this by using depthwise separable convolutions only in deeper layers, reducing trainable parameters and FLOPs.
- Many existing models are not trained from scratch and depend on pretrained resource-demanding architecture. These pretrained models are trained on datasets that differ from the AD dataset, which can introduce biases in the features extraction of grayscale medical images. In our research, deep neural networks are trained from scratch, enabling control over the training process and eliminating biases linked with pretrained weights.
- While existing AD classification models use oversampling techniques like SMOTE and SMOTE-Tomek to address class imbalance among four classes, this work employs the SMOTEENN (hybrid undersampling and oversampling) technique to handle class imbalance across five stages of AD: MCI, EMCI, LMCI, AD, and CN. SMOTEENN also addresses data overlapping issues and eliminates noise during data reconstruction.
- Most existing models do not emphasize skull stripping, a crucial step in medical image preprocessing. This works implements a skull stripping algorithm to ensure cleaner input data by removing unwanted non-brain features.
- Evaluation metrics such as accuracy, precision, F1 score, recall, and number of trainable parameters are compared with existing state-of-the-art (SOTA) deep learning models. To further assess the suggested model, the area under curve (AUC) value is calculated for each class. Existing works are summarized in Table 1.

## 3. Proposed Method

### 3.1. Dataset Collection

The Alzheimer’s Disease Neuroimaging Initiative (ADNI) repository contains the datasets for various classes of AD. The sample sizes are extensive, and all MRI, PET, and CT images are in DICOM (digital imaging and communications in medicine) and NIfTI (neuroimaging informatics technology initiative) format and only be viewed in a specific image viewer. The Kaggle dataset [34] used in this research contains preprocessed structural MRI images from the ADNI, with class label information. This dataset encompasses different classes, including CN, MCI, LMCI, EMCI, and AD. The images in this dataset are acquired from the Axial T2 STAR MRI neuroimaging sequence. The imaging protocol includes field strength = 3.0 tesla; manufacturer = SIEMENS; matrix (X = 256.0 pixels, Y = 256.0, Z = 44.0); Mfg model = I Prisma_fit; pixel spacing (X = 0.9 mm, Y = 0.9 mm); slice thickness = 4.0 mm; TE = 20 ms; TI = 0.0 ms; TR = 650.0 ms; and weighting = T2.

### 3.2. Preprocessing of Dataset

#### 3.2.1. Data Cleaning

Data were cross-validated against the original dataset using patient ID as the identifier, and discrepancies were resolved. During this process, we found that some images were misclassified between the AD and LMCI groups. Irrelevant images were then removed from the data in the MCI and CN classes. The images were resized into 224 ×224.

#### 3.2.2. Skull Stripping

**Figure 1:**
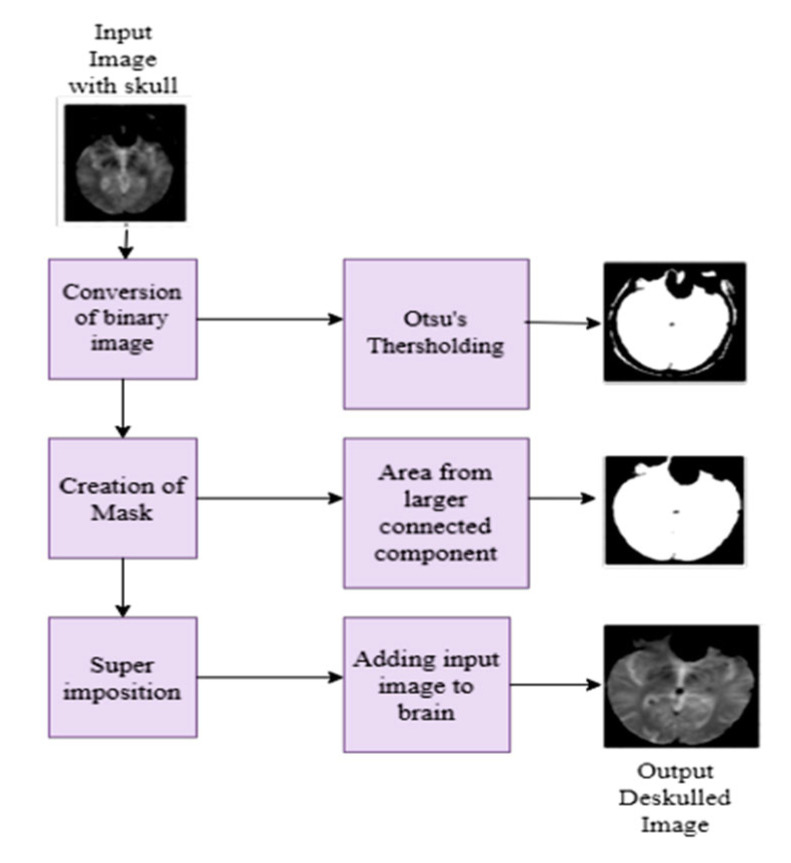
Workflow of the skull stripping algorithm.

**Figure 2:**
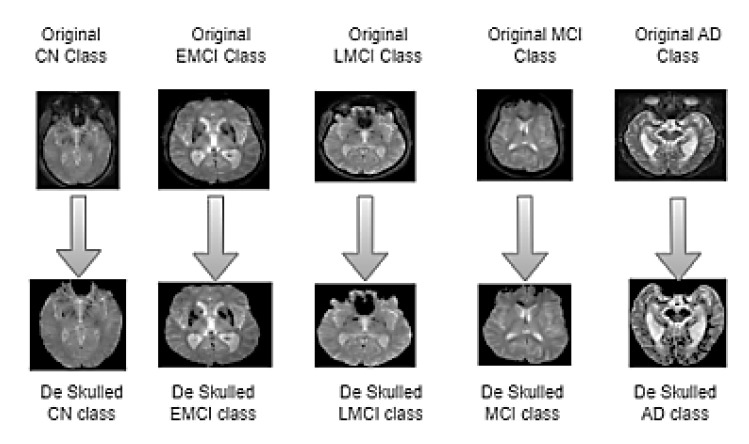
Skull stripped images of five classes. The images in the top row represent the original MRI slice from CN, EMCI, LMCI, MCI, and AD classes. The corresponding images in the bottom row illustrate the results of the skull stripping algorithm applied to each class.

In MRI images, presence of non-brain tissue (skull) can significantly increase computational time when extracting features from areas not of interest. To address this issue, a mathematical morphology model [35,36], including Otsu’s thresholding, is applied to strip the skull from the brain. This algorithm first converts the input image into a binary image and divides the pixels into background and foreground based on their intensity value. Subsequently, erosion and dilation operations are performed according to the structuring elements. The workflow of the brain stripping algorithm and deskulled images of five classes are depicted in Figure 1 and Figure 2, respectively. The dice coefficient is a statistical measure to compare the similarity between the predicted mask and the ground truth mask. Table 2 describes the measurement of the dice coefficient for different classes, which clarifies the accuracy of Otsu’s skull stripping algorithm. The dice coefficient is calculated from the predicted brain mask A and ground truth brain mask B, as in the following Equation (1):

(1) $$\mathrm{Dice}\;\mathrm{Coefficient}=\frac{2|A\cap B|}{\left|A|+|B\right|}$$

#### 3.2.3. Balancing Data in All Classes

Resampling approaches are intended to modify the class distribution in the training datasets. Once the class distributions have been more evenly distributed, the standard deep learning classification methods can successfully fit the changed datasets. In the minority class, oversampling creates new synthetic instances, whereas undersampling approaches eliminate or merge examples in the majority class. Both resampling techniques can be helpful but are more effective when used in combination. In this dataset, the number of images in all classes are not equal and it leads to a significant class imbalance problem. Utilizing the imbalanced dataset will be reflected by reducing the accuracy of the model.

To rectify the class imbalance problem in this dataset, the SMOTEENN [37,38] algorithm is implemented. SMOTE is the most often used oversampling technique, and it can be used with various undersampling approaches. The edited nearest neighbors, or ENN, rule is a prominent undersampling strategy. This approach involves utilizing k = 3 nearest neighbors to find misclassified cases in a dataset and remove them from the dataset. The selection of a k-value in the SMOTEEENN and its impact on the validation and testing accuracy is given in Table 3. The fundamental idea behind the SMOTE oversampling technique is to create new minority class examples by interpolating multiple minority class examples that lay close to one another. While it can significantly increase the model’s classification accuracy, it can also produce boundary and noise samples. ENN is utilized as a data cleaning technique that can eliminate any sample whose class label differs from the class of at least two of its three nearest neighbors and creates the samples. This will produce better defined class clusters. SMOTE+ENN lessens the chance of underfitting. It can be applied to all classes or just the majority class examples. The original dataset has 1220, 240, 72, 922, and 145 images in the classes of CN, EMCI, LMCI, MCI, and AD, respectively. In SMOTEENN, the number of images in the CN class is undersampled to 649, and the number of images in the EMCI, LMCI, MCI, and AD classes are oversampled to 1201, 1220, 961, and 1218, respectively.

### 3.3. Proposed Model Architecture

**Figure 3:**
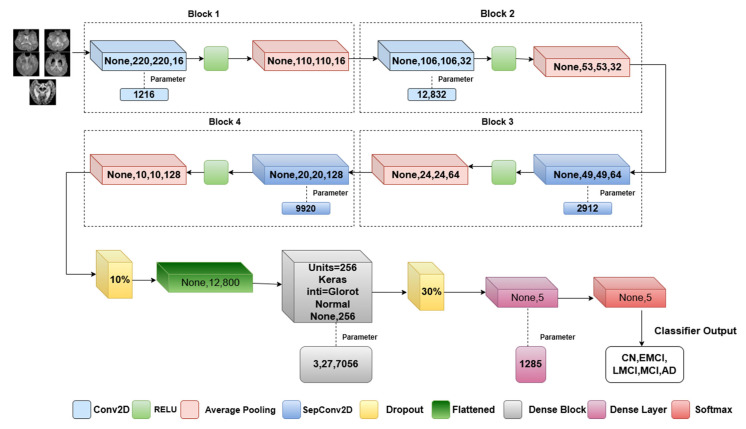
Proposed model architecture.

In the proposed method, convolutional mixer architecture is implemented to differentiate the features of MRI in the different phases of AD. This will leverage both separable convolutions and a standard convolution layer for efficient feature extraction. The architectural approach, as in Figure 3, differs in the usage of the separable convolution operation. The convolutional mixer uses multiple depthwise and pointwise convolutions with residual connections. Our model simplifies this by using **depthwise separable convolutions only in deeper layers**, reducing the **trainable parameters and FLOPs**, as reflected in our efficiency metrics. It consists of two depthwise separable convolutional layers with a ReLU nonlinear activation function where the glorot normal initializer is used to initialize the network weights (Algorithm 1).
**Algorithm 1.** describes the proposed methodologyInput: X^224 × 224 × 1^(Grayscale brain Images)
**Step 1: Data preprocessing**
Normalize pixel value [0, 1]
Data augmentation-SMOTEENN
Train_Validation_Test split
**Step 2: Implementation of Convolutional mixer**
for each block in [1, 2]:
Conv2D(I, filters = 16*block, kernel_size = 5× 5, activation = ReLU)
AveragePooling2D(x, pool_size = 2 × 2)
for each depth_block in [1, 2]:
if depth_block == 1:
DepthwiseSeparableConv(x, kernel = 5 × 5, filters = 64, activation = ReLU)
else:
DepthwiseSeparableConv(x, kernel = 5× 5, filters = 128, activation = ReLU)
AveragePooling2D(x, pool_size = 2 × 2)
**Step3: Classification Layer**
# Apply Dropout and Flatten
Dropout(x, rate = 0.5)
Flatten(x)
# Fully Connected Layers
for i = 1 to 2:
If i == 1:
Dense(x, units = 256, activation = ReLU)
Dense(x, units = 5)
# Output Layer (Softmax activation)
Softmax(x)
**Step4: Training Process**
#Model compile
Optimizer-Adam,Epochs-50,Learning rate-0.01,Batch size-8.
**Step 5: Evaluation Metrics**
Plotting of Training,Validation-Accuracy,Recall,Loss,Precision,F1 Score,ROC plot,AUC.

#### 3.3.1. Convolutional Layer

The low-level features are extracted from the first two standard convolutional layers. It has a 5 × 5 convolution layer with 16 filters followed by another 5 × 5 convolutional layer with 32 filters. The number of parameters is calculated as in Equation (2) and the dimension is calculated as in Equation (3). In these equations, *H* is the input feature map height, *W* is the input feature map width, I is the input volume, F is the number of filter, *P* is the padding, and *S* is the stride specifies the step size of kernel movement. The convolution operation is performed as in Equation (4).

(2) Number of parameters=(H ∗ W ∗ I+bias) ∗ F

(3) $$Output\;width=(\left(I-F\;+\;2\;*\;P\right)/S$$

(4) $$conv{\left(W,Y\right)}_{a,b}=\sum_{l,m}^{L,M}W_{l,m}Y_{a+l,b+m}$$

A convolutional layer shifted the kernel W at position l,*m* (indicies for the kernel *W*) of the filter over the input feature map *Y* to compute the weighted sum of the feature values at each position, where the indices *a,b* is the output feature map to store the result of the convolutional operation.

#### 3.3.2. Separable Convolutional Layer

Depthwise separable convolution separates the spatial and channel wise computations, greatly minimizing the number of parameters and FLOPs (floating-point operations per second). The depthwise convolution applies a spatial filter to each input channel separately. Each channel has its own filter, performing only the filtering operation without combining channels, as in the following Equation (5):

(5) $$Depthwise\;conv{\left(W,Y\right)}_{a,b}=\sum_{l,m}^{L,M}W_{l,m}Y_{a,b,n}$$

where $W_{\left(l,m\right)}$ is the filter depthwise convolution and *Y* is the input feature map. The second operation, which only works on single “points”, is frequently called a “Point wise convolution” since it employs a kernel of size 1 × 1, representing no spatial interactions. The output of depthwise convolution is combined in this layer and produces new features, as described in Equation (6) as follows:

(6) $$Pointwise\;conv{\left(W,Y\right)}_{a,b}=\sum_{n}^{N}W_{n}Y_{a,b,n}$$

where $W_{n}$ is the depthwise filter specific to channel, $Y_{a,b,n}$ is the input channel tensor with indices (*a*, *b*) which represents the spatial dimensions, and *n* is the number of channels. The separable convolution is the concatenation of depthwise and pointwise convolution, as in Equation (7) which follows:

(7) $$Separable\;conv{\left(W_{pointwise},W_{Depthwise},Y\right)}_{a,b}=\;{Pointwise\;conv}_{\left(a,b\right)}(W_{pointwise},{Depthwise}_{\left(a,b\right),}\left(W_{Depthwise},Y\right))$$

#### 3.3.3. Activation and Pooling Layer

A nonlinear ReLU activation function criticizes the vanishing gradient problem. This function returns zero in the event of a negative input and returns any value x if it receives a positive input. It requires only the maximum function of 0 to x, as in Equation (8). Average pooling reduces the spatial dimension while selecting the features. Each layer is directly connected to the next layer without any pathways for the gradient flow.

(8) $$f\left(x\right)=max(0,x)$$

#### 3.3.4. Dropout and FCN

To fit the model to the dataset, a dropout layer is added to drop some nodes randomly at each epoch. The flatten layer converts the 2D data into one-dimensional data. The dense layer is the fully convolutional layer that takes the feature vectors from the flatten layer and produces the output. The multiclass labels are assigned to one hot encoding method to predict the class labels.

#### 3.3.5. Loss Function

The categorical loss function predicts the difference between the actual and predicted image class. Cross-entropy loss is given in Equation (9), in which  y is the actual value and $\hat{y}$ is the neural network predicted value for each class. This can be seen as follows:

(9) $$LF=-[y_{AD}\log\hat{y_{AD}}+y_{EMCI}\log\hat{y_{EMCI}}+y_{LMCI}\log\hat{y_{LMCI}}+y_{MCI}\log\hat{y_{MCI}}+\;y_{CN}\log\hat{y_{CN}}]$$

## 4. Ablation Study

The robustness of the proposed model is ensured in this ablation study. The elements considered for this study are the weight initializer, the pooling layer, and optimizers.

### 4.1. Altering the Weight Initializer

To ensure a model’s high accuracy, it is essential to appropriately initialize weights when creating and training the neural network. Improper weight initialization can lead to the vanishing and exploding gradient problem. The weights of a neural network can be customized during the training phase. It must, however, be initialized before the training phase of the model, and this initialization significantly impacts on network training. The limit value of initialization is based on $n_{in}$ (number of input connection) and $n_{out}$ (number of output connection).

#### 4.1.1. Performance of Normal Initialization

**Figure 4:**
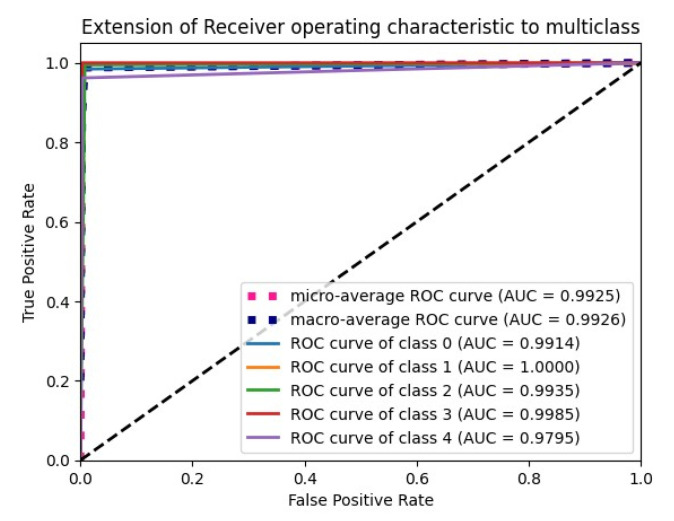
ROC plot for the Glorot normal initialization with average pooling and an Adam optimizer.

The “Glorot normal” initializer samples the weights at the truncated normal distribution (Gaussian) centered at zero and aims to equalize the variance between the input unit and output unit, even while achieving equality is unfeasible. Hence, variance is calculated as the average of the number of input and output units, as in Equation (10) below. According to Table 4, it performs well for the Adam optimizer with average pooling and the extension of it ROC plot is given in Figure 4.

(10) $$LimitValue=\sqrt{\frac{2}{n_{in}+n_{out}}}$$

**Figure 5:**
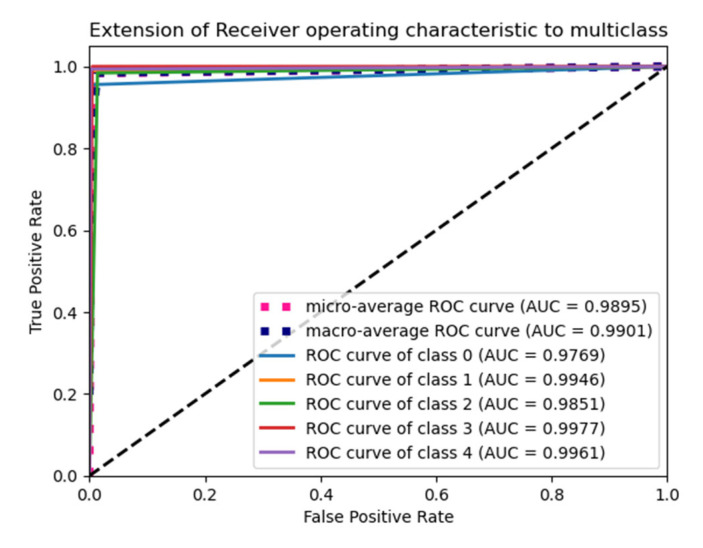
ROC plot for the He Normal initialization with average pooling and an Adam optimizer.

The “He Normal” initializer provides more effective convergence to the global minimum of the cost function by initializing the weights with a consideration of the size of the preceding layer. Despite still being random, the weights’ range varies based on the number of neurons in the preceding layer. It draws samples from a truncated normal distribution centered on 0, and its standard deviation is given in Equation (11) below. From Table 5, it performs well for the Nadam optimizer with average pooling, and its ROC plot is depicted in Figure 5.

(11) $$Limit\;value=\sqrt{\frac{2}{n_{in}}}$$

#### 4.1.2. Performance of Uniform Initialization

**Figure 6:**
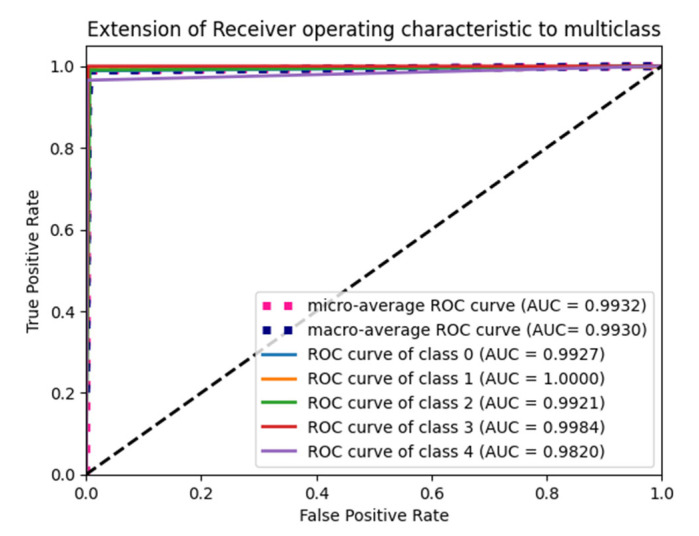
ROC Plot for the Glorot uniform initialization with max pooling and an Adam optimizer.

In uniform initialization, the initial value is created using a uniform distribution with a maximum value of 1 and a minimum value of −1. The range of the uniform distribution varies depending on the number of input units and output units for each layer. The Glorot uniform initializer limit value is given in Equation (12). An Adam optimizer with a max pooling operation gives the highest testing and validation accuracy in Table 6, and its ROC plot is given in Figure 6.

(12) $$Limit\;value=\sqrt{\frac{6}{n_{in}+n_{out}}}$$

**Figure 7:**
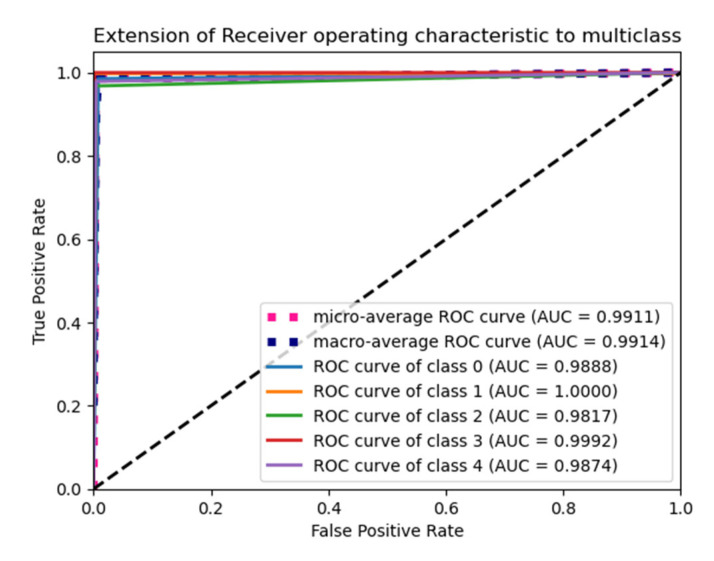
ROC plot for the He Uniform initialization with average pooling and an Adam optimizer.

The weights used in the “He Uniform” initialization method have a normal distribution, with a mean of zero and a standard deviation as shown in Equation (13). An Adam optimizer with average pooling gives the highest accuracy in Table 7, and its ROC plot is shown in Figure 7.

(13) $$Limit\;value=\sqrt{\frac{6}{n_{in}}}$$

### 4.2. Altering Pooling Operation

Pooling layers produce the downsampled representation of features in the input. It is important to consider that slight changes in the feature’s location recognized by the convolutional layer in the input still results in a similar output with the pooled feature map. The model’s invariance to local translation is added by the pooling operation. Max pooling and average pooling are the two pooling layers that can be added after the nonlinear activation function. Max pooling chooses the maximum intensity of a pixel. It is formulated as in Equation (14), which is as follows:

(14) $$Maxpooling\;2D(D)a,\;b\;,\;c=\underset{x,y}{\max}D_{a.q_{i}+x,b.q_{j}+y,c}$$

where D is the input and number of channel *c*, *q_i_* is the stride of the row, and *q_j_* is the stride of the column. Average pooling takes the average number of pixels. It is formulated as in Equation (15), which is as follows:

(15) $$Averagepooling2D(D)a,\;b\;,\;c=\frac{1}{f_{i\;\;\;}f_{j}}\sum_{x,y}D_{a.q_{i}+x,b.q_{j}+y,c}$$

### 4.3. Altering the Optimizers

The optimizers are tested with a default learning rate. Adam moment estimation gives the highest accuracy. Testing loss is lower, and the prediction of true positive and false positive rates is high in Adam. Hence, Adam gives the best evaluation metrics compared to Adagrad, Adamax, and Nadam.

### 4.4. Parameter Selection Based on Ablation Study

In Table 4, a glorot normal initializer with an Adam optimizer and average pooling efficiently trains the neural network with high performance metrics. In Table 5, a Nadam optimizer with average pooling and an Adam optimizer with max pooling both give the highest testing accuracy compared to glorot normal, but the testing and validation accuracy is mismatched. In Table 6, a glorot uniform weight initializer’s performance is less when compared to glorot normal with all types of optimizers and pooling. In Table 7, the uniform initializer with an adam optimizer and max pooling gives the highest testing accuracy compared to glorot normal. But, validation accuracy is lower and testing loss is high. Hence, a glorot normal initializer with an Adam optimizer and average pooling operation is used to train and evaluate the proposed model.

## 5. Performance Evaluation Metrics

To evaluate the model, the parameters predicted from the multiclass (five class) confusion matrix are: true negative (TN: number of images truly classified as false), true positive (TP: number of images truly classified as true), false positive (FP: number of images wrongly classified as true), and false negative (FN: number of images wrongly classified as false).

A receiver operating characteristic curve, or ROC curve, is a graph that displays how well a classification model performs. It enables us to identify the model classification at various levels of certainty. The curve is divided into two lines: one for how frequently the model identifies positive cases (true positives) and another for how often it incorrectly labels negative cases as positive (false positives). The selection of the model threshold can be identified from the graph.

The AUC quantifies the likelihood that the model will assign a higher projected probability to a randomly selected positive case than a randomly chosen negative instance. Other metrics include accuracy, precision, recall, and F1 score [39].

The accuracy is the number of successfully categorized images divided by the total number of images, as given in Equation (16). Precision in Equation (17) is the measure of perfectly predicted images turned into positives. Therefore, it can be used as an evaluation metric where the false positives are higher than the false negatives. Recall, as shown in Equation (18), measures the number of actual positive images correctly predicted. It can be used as the evaluation metric of the model when the false negatives dominate the false positives. It is the rate of true positives or the sensitivity of the model.

(16) $$Accuracy=\frac{TP+TN}{TP+TN+FP+FN}$$

(17) $$Precision=\frac{TP}{TP+FP}$$

(18) $$Recall=\frac{TP}{TP+FN}$$

The harmonic mean of precision and recall is the *F1* score. If precision increases, then recall decreases and vice versa. The *F1* score, as shown in Equation (19), captures both precision and recall in a single value. It is also known as the dice coefficient.

(19) $$F1\;Score=\frac{2TP}{2TP+FP+FN}$$

## 6. Results and Discussion

### 6.1. Experimental Setup

The proposed algorithm is performed on an NVIDIA Quadro RTX4000 with 24 GB RAM, utilizing the Jupyter Notebook version 7.0.3 in the Anaconda environment. The layers are implemented with a Keras and Tensorflow backend. Model evaluation parameters are tested using Scikit-learn and Numpy. The dataset is split into 80% and 20% for training and validation. The learning rate, batch size, and number of epochs are selected using the grid search algorithm. The hyperparameter used in this model is of 50 epochs, a batch size of 8, and 0.001 as an initial learning rate, and an Adam optimizer is used to train the model, as described in Table 8.

### 6.2. Accuracy and Loss

**Figure 8:**
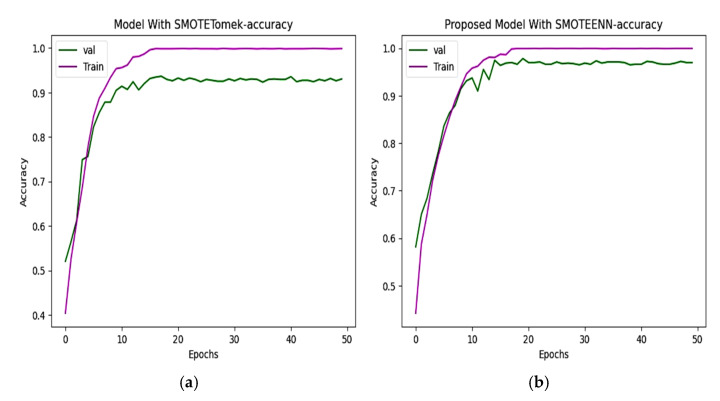
(**a**) CNN with SMOTETomek. This model achieves high training accuracy, and the validation accuracy saturated earlier which represents overfitting. (**b**) Proposed model with SMOTEENN. The training and validation accuracy improve rapidly, which effectively handles class imbalance.

**Figure 9:**
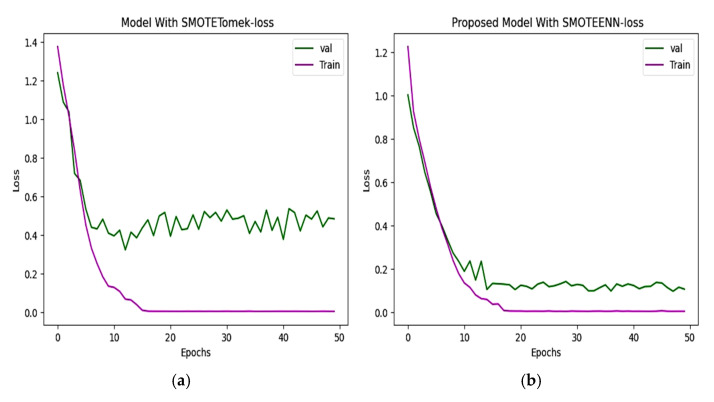
(**a**) Loss progression of CNN with SMOTETomek shows a steep decline in training loss and validation loss, with noticeable fluctuations indicating suboptimal learning. (**b**) Loss progression of the proposed model shows a consistent decline in the training and validation loss, reflecting effective learning of unseen data.

Two hybrid sampling techniques, SMOTETomek and SMOTEENN, are compared using then Kaggle–ADNI dataset. In Figure 8, the proposed model offers an accuracy of 98.80%. In Figure 9, the predicted classes of images varied from the ground truth image with a loss of 0.075352, which is less than the 0.3331 of SMOTETomek. The validation loss for the CNN model is 0.3280 and the validation loss for the proposed work is 0.08963.

### 6.3. Precision and Recall

**Figure 10:**
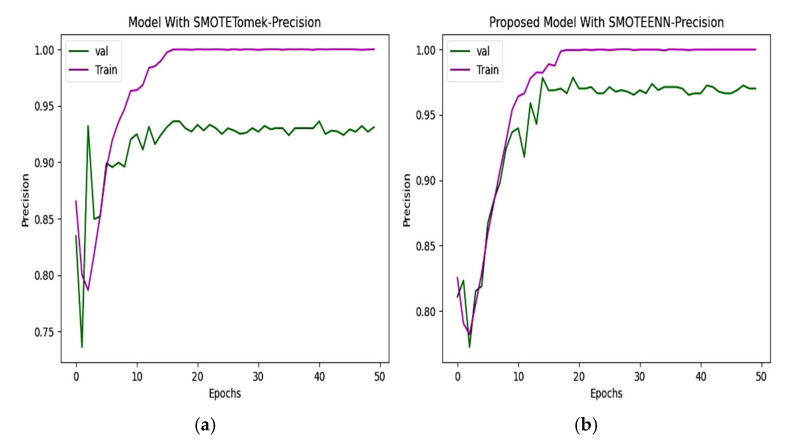
Precision of (**a**) CNN with SMOTETomek. The training precision quickly reaches near-perfect levels, and the validation precision fluctuates in the initial epochs and stabilizes slightly below that of the SMOTEENN-enhanced model. (**b**) The training precision rapidly increases, while the validation precision shows a steady upward trend, maintaining values above 95% after the 10th epoch in the proposed model.

**Figure 11:**
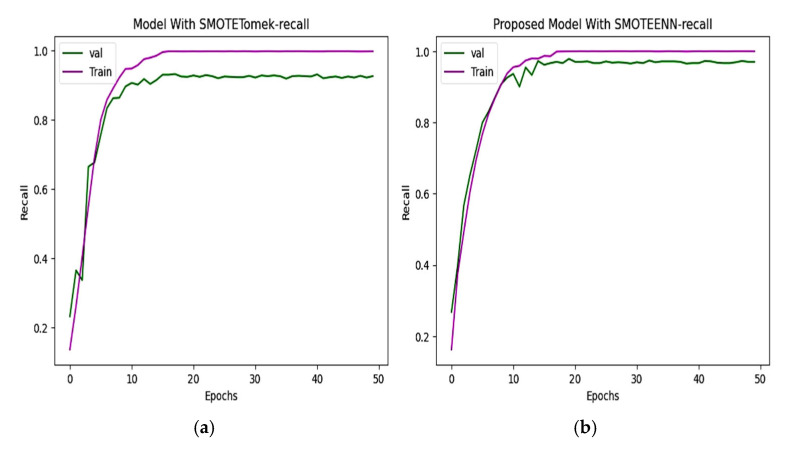
Recall of (**a**) CNN with SMOTETomek. The training recall reaches nearly perfect values, and the validation recall stabilizes slightly lower, just below 0.9. (**b**) The proposed model with SMOTEENN shows close alignment between training and validation recall, which suggests strong generalization ability and a well-balanced learning process.

The precision and recall of the CNN and proposed model are depicted in Figure 10 and Figure 11. The precision of the CNN model is 93.49%, whereas for the proposed work it is 98.80%. The recall of the proposed model is 98.68%, which is greater than the recall of the CNN model at 92.72%.

### 6.4. F1 Score and ROC Plot

**Figure 12:**
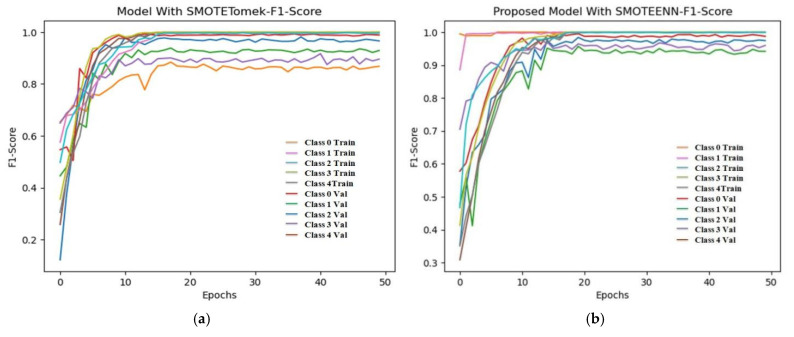
Training and validation F1 score of (**a**) CNN with SMOTETomek and (**b**) proposed model with SMOTEENN.

**Figure 13:**
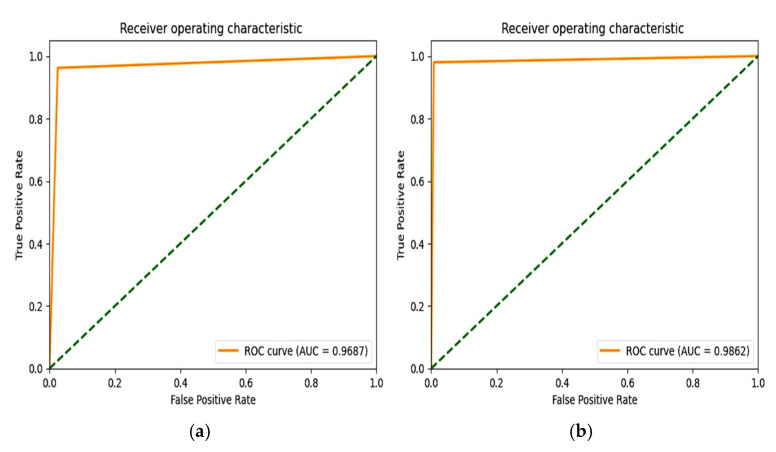
ROC plot of (**a**) CNN with SMOTETomek (**b**) and proposed model with SMOTEENN.

**Figure 14:**
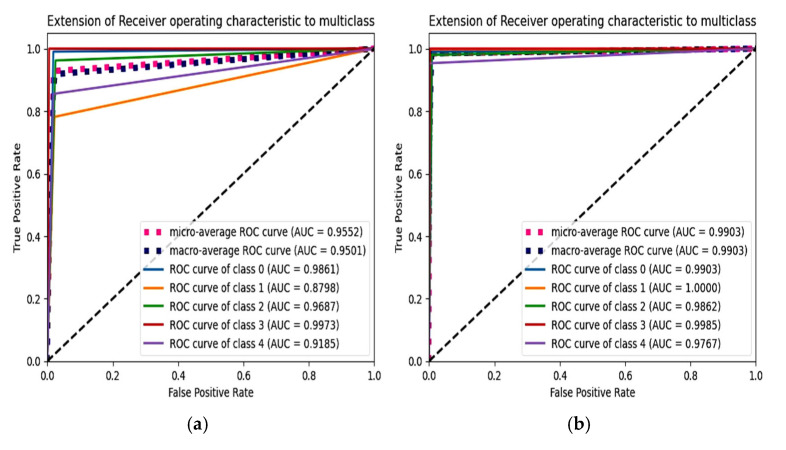
Extension of ROC plot for each class. (**a**) CNN with SMOTETomek and (**b**) proposed model with SMOTEENN.

From Figure 12, the F1 score of the CNN is 92.84% and the proposed model achieves a score of 98.86%. An extension of the ROC plot of one class versus other classes and the ROC plot are given in Figure 13 and Figure 14. It is proved that all the classes are classified with high AUC (area under curve) in the proposed model, which is higher than that of the CNN model.

### 6.5. Confusion Matrix and Trainable Parameter

**Figure 15:**
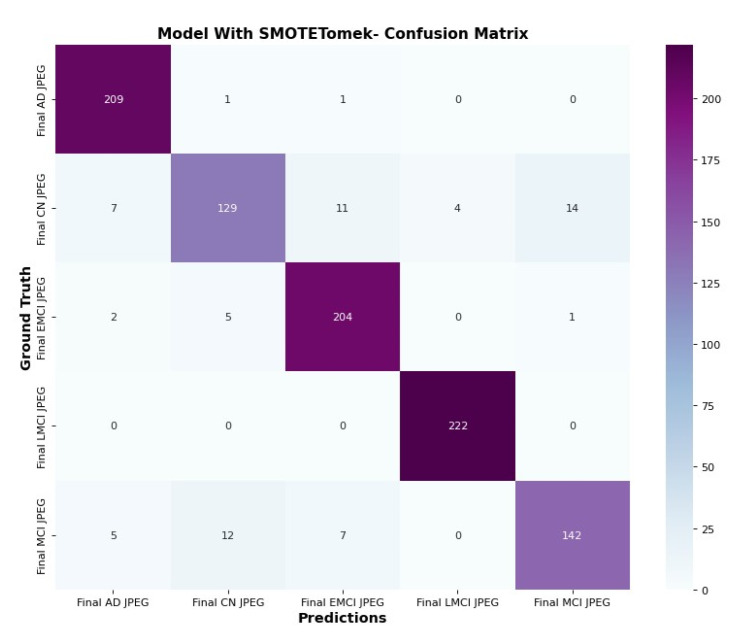
Confusion matrix of CNN with SMOTETomek.

**Figure 16:**
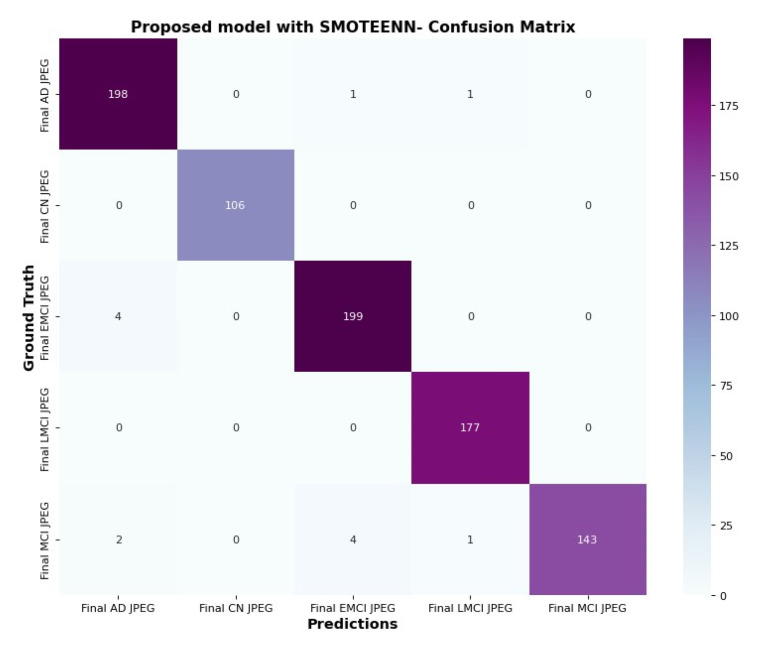
Confusion matrix of proposed model with SMOTEENN.

The confusion matrix in Figure 15 and Figure 16 clarifies the correct prediction of images across various classes in the datasets. The proposed architecture reduces the number of trainable parameters compared to the CNN model with the SMOTETOMEK model.

### 6.6. Visualization of ASOP

**Figure 17:**
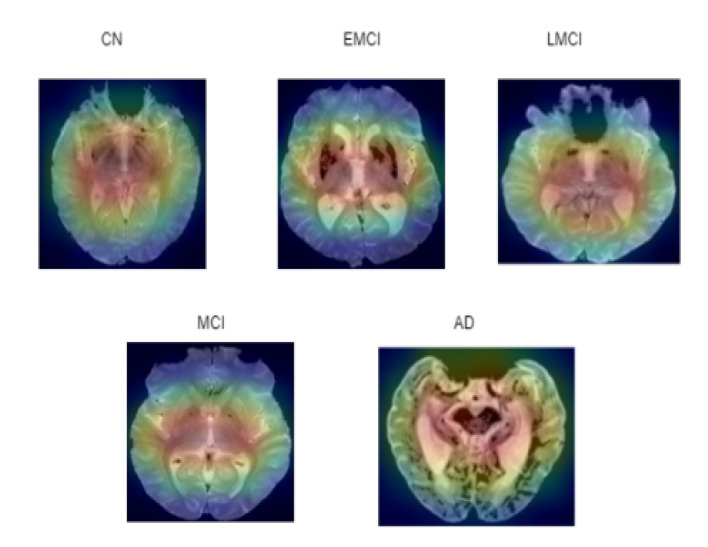
ASOP of five classes where red indicates region of higher importance to the model’s decision making, while green and blue represent regions of lower importance.

Numerous studies have been conducted to help deep learning become more intelligible and useful. Additionally, it is essential to improve the comprehension of deep neural networks in multiple deep learning applications related to medical imaging. Deep learning is demonstrated by an ASOP [40]. ASOP provides a visual representation of any highly connected neural network. This aids in learning more specifics about the model and highlights the affected areas of the brain according to the different stages of AD. It is applied to the final convolution layer after obtaining the predicted label using the indicated model, as shown in Figure 17. In Equation (20), the activation maps $A^{k}$ and gradients G are captured from the last convolutional layer *L*. The gradients $\frac{\partial y^{c}}{\partial A^{k}}$ are computed with respect to the predicted class score $y^{c}$. The important weights $\alpha_{k}$ are computed using global average pooling over the gradients. The ReLU operation visualized only the positive values. The final ASOP S is upsampled to match the original image size and overlaid for interpretation.

(20) $${ASOP}_{L}=ReLU(\sum_{k}\alpha_{k}A^{k\;})$$

## 7. Discussion

### 7.1. Interpretation

This work aims to model a deep neural network to classify the progression of AD. The convolutional layer extracts the features from structural MRI, while the stacking of separable convolution layers reduces computational complexity and the number of trainable parameters. In our work, the trainable parameters for training the network are less than Mobilenet [20], which uses the same dataset. The proposed model is compared with an existing CNN model that uses the SMOTETomek [36] sampling technique to rectify the class imbalance problem. In the SMOTETomek hybrid approach, Tomek links find and eliminate data from the majority class that are strongly linked to the minority class, while SMOTE creates synthetic data for the minority class. Our work with SMOTEENN improves the reconstructed data in both majority and minority classes. The existing model and state-of-the-art models are analyzed with the same dataset, and its results are discussed in Table 9. The main objective of this comparative study is to analyze the tradeoff between the proposed model and existing model. The trainable parameters of MobileNet are closer to the proposed model, despite it giving an accuracy of 88%. ResNet151 achieves an accuracy of 91% despite having a higher number of trainable parameters compared to MobileNet. Models such as VGG19 and ResNet 152, while effective, are not suitable for devices with limited memory. The proposed model achieves 98.87% accuracy with only 3.3 M trainable parameters and lower FLOPs compared to conventional deep learning architectures like Resnet and VGG. It reduces the computational complexity and also the training time of the model.

### 7.2. Implication

The improved precision and effectiveness of the proposed model can significantly enhance the early detection of Alzheimer’s disease (AD). Early detection leads to better patient outcomes and enables more personalized treatment regimens, potentially improving the quality of life for patients. The reduction in trainable parameters without sacrificing accuracy highlights the efficiency of the proposed model. This could lead to faster and more cost-effective AD classification, making it more accessible for clinical use. The success of the SMOTEENN approach in improving data reconstruction suggests further exploration of advanced sampling techniques to enhance model performance in other medical imaging and classification tasks.

### 7.3. Strength

Models with separable convolutions are typically better than ordinary convolutions, producing more accurate models with fewer parameters and reduced computational complexity. The SMOTEENN for data preprocessing not only resolved the data imbalance issue but also addressed the SMOTE algorithm’s susceptibility to noise and overlapping data.

### 7.4. Limitations

The datasets from other data sources have different sample distribution in feature space; hence, this model cannot be employed to achieve the exact evaluation metrics for real time data. SMOTEENN involves generating synthetic samples and cleaning noisy samples, which requires expensive computational resources for larger datasets. The classification task solely focuses on features in the different stages of disease, and demographic details are excluded. The images in the dataset are collected exclusively from the Axial T2 STAR MRI neuroimaging sequence. Compared to a 2D CNN model, a 3D CNN model can extract more features from medical images.

## 8. Conclusions

The five classes of AD are classified with a validation accuracy of 98.92%, a validation recall of 98.80%, a validation loss of 0.0863, a validation F1 score of 98.93%, a testing accuracy of 98.81%, a testing recall of 98.69%, a testing loss of 0.0724, and a testing F1 score of 98.86%, which is higher than that of the model with SMOTETomek. These outstanding results show the impact of our approach in leveraging deep learning for the accurate and reliable classification of Alzheimer’s disease stages from structural MRI scans. The use of separable convolution in the hidden layers of the deep neural network reduces the trainable parameters without compromising classification accuracy. A brain stripping algorithm removes the skull from MRI images, which solely focus on the brain tissue. The SMOTEENN sampling process effectively generates synthetic samples and reduces the noisy samples by the K nearest neighbor verification process, which balances the datasets based on features in different classes of images. Additionally, the ASOP technique provides insight into the learning of relevant features associated with each class and enhances the interpretability of the proposed model.

## 9. Future Work

In the future, image fusion techniques will be implemented to explore information from different imaging modalities. This model can be applied to classify progressive and stable MCI stages of AD. Volumetric analysis should be embedded to predict atrophy in the brain regions. The OASIS and NAAC datasets will be used to validate the model’s performance. Artifacts in CT/PET and MRI images will be considered, and the image preprocessing techniques will be optimized. Clinical data and demographic details will be included in future to improve the effectiveness of treatment. Future research will explore the integration of vision transformer models to evaluate their impact on the performance of the proposed method.

## Figures and Tables

**Table 1:** Summary of survey.

Year	Technique	Dataset	Classification	Model Accuracy %	Imbalance Handling
2024	EfficientNetB0 [11]	ADNI	2-way	98.94	-
2024	CNN [12]	ADNI	4-way	97.5%	Data augmentation
2024	STCNN [13]	OASIS-kaggle	4-way	99.36%	SMOTETomek
2023	Modified Inception V3 [14]	ADNI	6-way	98.67	Data augmentation
2023	SCNN [15]	ADNI	4-way	91.83	-
		OASIS		93.85	-
2023	Resnet50v2 [16]	ADNI	5-way	91.84	-
2023	Mobile Net [17]	Private Dataset	5-way	96.6	-
2023	DeepCurvelet convolutional [18]	ADNI-kaggle		98.6	-
2022	Modified CNN-DEMNET [19]	ADNI	4-way	84.83	SMOTE
2022	Modified CNNADD-NET [20]	Kaggle-OASIS	4-way	98.63	SMOTETomek
2022	VGG19 [21]	ADNI	4-way	97	-
2022	LeNet [22]	ADNI	2-way	83.7	-
2022	DCNN [23]	ADNI	2-way	95.2	-
2022	MCNN [24]	ADNI	AD and CN	97.78	-
			pMCI and MCI	79.90	-
2021	CNN with inception [25]	ADNI	3-way	94.9	-
2021	Alexnet [24]	ADNI	4-way	99.61	-

**Table 2:** Dice coefficient.

Class	Dice Coefficient
AD	96.1819
CN	94.2001
LMCI	96.8123
EMCI	95.612
MCI	93.457
Overall	**95.257**

**Table 3:** Selection of k-value.

K-Value	Validation_Accuracy	Testing_Accuracy
3	98.92	98.81
5	61.86	61.86
7	56.08	56.08

**Table 4:** Performance analysis of Glorot normal.

Optimizer	Pooling	Validation Accuracy	Testing Accuracy	Validation Loss	Testing Loss	Validation F1Score	Testing F1Score
Adam	Max Pooling	98.32	98.68	0.1045	0.0707	98.34	98.76
	Average Pooling	98.92	98.81	0.0863	0.0724	98.93	98.86
Adagrad	Max Pooling	59.74	65.39	0.9454	0.8834	63.52	68.69
	Average Pooling	59.74	65.39	0.9454	0.8834	63.52	68.69
Adamax	Max Pooling	98.32	98.21	0.1002	0.0956	98.27	98.36
	Average Pooling	97.72	98.09	0.1018	0.0751	97.63	98.19
Nadam	Max Pooling	98.32	97.85	0.0539	0.1211	98.43	98.03
	Average Pooling	92.01	92.62	0.3819	0.3766	91.88	92.65

**Table 5:** Performance analysis of He Normal.

Optimizer	Pooling	Validation Accuracy	Testing Accuracy	Validation Loss	Testing Loss	Validation F1Score	Testing F1Score
Adam	Max Pooling	98.24	99.18	0.0747	0.0168	98.22	99.18
	Average Pooling	97.84	98.44	0.1308	0.1544	98.02	98.59
Adagrad	Max Pooling	93.99	71.60	0.7248	0.7086	75.13	74.02
	Average Pooling	70.19	71.89	0.7373	0.7053	72.47	74.02
Adamax	Max Pooling	98.20	97.96	0.0896	0,1047	98.25	98.10
	Average Pooling	98.80	98.57	0.0811	0.0769	98.85	98.61
Nadam	Max Pooling	97.96	98.80	0.1282	0.0378	98.04	98.91
	Average Pooling	99.52	98.92	0.0356	0.0514	99.54	98.92

**Table 6:** Performance analysis of Glorot uniform.

Optimizer	Pooling	Validation	Testing	Validation	Testing	Validation	Testing
		Accuracy	Accuracy	Loss	Loss	F1Score	F1Score
Adam	Max Pooling	98.80	98.91	0.1011	0.0402	98.92	98.97
	Average Pooling	98.44	97.84	0.0998	0.1045	98.39	97.98
Adagrad	Max pooling	59.98	62.96	0.9730	0.9572	63.47	66.04
	Average Pooling	64.90	66.94	0.8655	0.8492	69.09	70.05
Adamax	Max pooling	98.56	98.69	0.0899	0.1029	98.55	98.71
	Average Pooling	98.32	97.96	0.1253	0.1342	98.42	97.88
Nadam	Max pooling	98.80	97.84	0.0715	0.1591	98.84	97.81
	Average Pooling	97.38	98.33	0.1815	0.0860	97.56	98.45

**Table 7:** Performance analysis of He Uniform.

Optimizer	Pooling	Validation Accuracy	Testing Accuracy	Validation Loss	Testing Loss	Validation F1Score	Testing F1Score
Adam	Max Pooling	98.80	98.09	0.0515	0.1581	98.77	98.09
	Average Pooling	99.16	98.45	0.0516	0.1371	99.20	98.59
Adagrad	Max Pooling	78.25	79.67	0.6275	0.5887	80.75	81.37
	Average Pooling	70.19	72.23	0.7936	0.7878	73.43	74.96
Adamax	Max Pooling	97.96	99.04	0.1031	0.07004	98.02	99.00
	Average Pooling	98.20	98.44	0.0940	0.12104	98.21	98.52
Nadam	Max Pooling	98.65	98.67	0.0715	0.04966	98.67	98.65
	Average Pooling	97.36	98.19	0.1551	0.0719	97.42	98.16

**Table 8:** Hyperparameter selection for model training.

Parameter	Selection Value	Optimal Value
Learning rate	(0.01, 0.001, 0.00001)	0.01
Batch size	(8 or 16)	8
Epochs	(10–50)	50

**Table 9:** Comparison of proposed model with existing and state-of-the-art models.

Models	Accuracy %	Precision %	Recall %	F1 Score %	Trainable Parameter	FLOPS (M-Million and B-Billion)	Computation Time(s)
Mobile Net [14]	96.0	96.0	96.0	96.0	25,958,917	-	-
Existing model with SMOTETOMEK	92.82	93.49	92.72	92.84	3,548,581	717.59 M	75.28
DenseNet201	91.0	90.0	89.0	89.0	18,926,389	4 B	419.50
VGG19	89.0	90.0	86.0	88.0	20,188,229	19.6 B	2056.42
Mobile Net	88.0	87.0	84.0	85.0	3,556,349	314 M	32.96
ResNet152	91.0	90.0	89.0	89.0	59,026,309	11 B	1153.94
Inception V3	90.0	85.0	87.0	86.0	22,171,429	5.7 B	597.40
Xception	85.0	87.0	82.0	84.0	21,516,845	8.4 B	880.49
EfficientNetV2S	90.0	89.0	87.0	88.0	20,740,965	8.4 B	888.89
Proposed Model without SMOTEENN	70.19	77.81	66.59	41.93	3,305,221	126.4 M	13.28
Proposed Model with SMOTEENN	98.87	98.80	98.60	98.86			

## Data Availability

Data used in this research is referenced [27].
